# A retrospective analysis of the prognosis of Japanese patients with sarcoma brain metastasis

**DOI:** 10.1002/cam4.5710

**Published:** 2023-02-22

**Authors:** Yu Toda, Eisuke Kobayashi, Daisuke Kubota, Yasuji Miyakita, Yoshitaka Narita, Akira Kawai

**Affiliations:** ^1^ Department of Musculoskeletal Oncology National Cancer Center Hospital Tokyo Japan; ^2^ Department of Medicine for Orthopaedics and Motor Organ Juntendo University School of Medicine Tokyo Japan; ^3^ Department of Neurosurgery and Neuro‐Oncology National Cancer Center Hospital Tokyo Japan

**Keywords:** alveolar soft part sarcoma, brain metastasis, sarcoma, stereotactic radiosurgery, sunitinib

## Abstract

**Background:**

Bone and soft tissue sarcomas are rare tumors and extremely rarely metastasize to the brain. Previous studies have examined the characteristics and poor prognostic factors in cases of sarcoma brain metastasis (BM). Due to the rarity of cases of BM from sarcoma, limited data exist concerning the prognostic factors and treatment strategies.

**Methods:**

A retrospective single‐center study was performed on sarcoma patients with BM. The clinicopathological characteristics and treatment options for BM of sarcoma were investigated to identify predictive prognostic factors.

**Results:**

Between 2006 and 2021, 32 patients treated for newly diagnosed BM at our hospital were retrieved among 3133 bone and soft tissue sarcoma patients via our database. The most common symptom was headache (34%), and the most common histological subtypes were alveolar soft part sarcoma (ASPS) and undifferentiated pleomorphic sarcoma (25%). Non‐ASPS (*p* = 0.022), presence of lung metastasis (*p* = 0.046), a short duration between initial metastasis, and the diagnosis of brain metastasis (*p* = 0.020), and the absence of stereotactic radiosurgery for BM (*p* = 0.0094) were significantly correlated with a poor prognosis.

**Conclusions:**

In conclusion, the prognosis of patients with brain metastases of sarcomas is still dismal, but it is necessary to be aware of the factors associated with a relatively favorable prognosis and to select treatment options appropriately.

## Graphical abstract

We retrieved Japanese sarcoma patients with brain metastasis (BM) in a single institution and analyzed the prognostic factors for BM. We have identified several prognostic factors for brain metastases. We believed that our data is useful for understanding the prognosis and treatment strategy for BM of sarcoma.

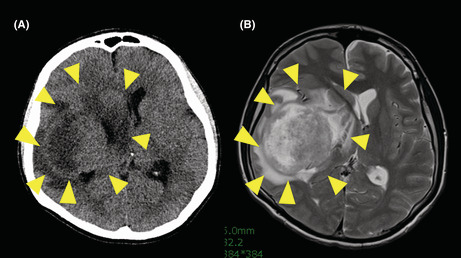

## 1 INTRODUCTION

Soft tissue and bone sarcomas are rare tumors among malignancies and are classified into more than 50 histological subtypes. Although sarcomas typically have the potential to spread to the lungs as well as the liver and bone, brain metastasis (BM) from sarcoma is much rarer.^1^, ^2^, ^3^, ^4^, ^5^, ^6^, ^7^, ^8^, ^9^, ^10^, ^11^, ^12^, ^13^, ^14^ The incidence of BM was estimated to be 0.6%–3.2% among sarcoma patients according to previous literature.^3^, ^4^, ^6^, ^12^, ^15^ In addition, the prognosis after BM is reportedly dismal.^1^, ^2^, ^3^, ^5^, ^7^, ^12^, ^14^

In general, metastasectomy, whole brain radiation therapy (WBRT), and stereotactic radiosurgery (SRS) are the treatment options for BM. SRS reduces radiation exposure to normal brain tissue compared to WBRT. The effectiveness of radiotherapy depends on the type of cancer in the primary tumor. Although no significant difference in overall survival for BM from lung cancer has been reported between WBRT and SRS,^16^ there are no reports comparing the outcomes of WBRT and SRS for BM from sarcomas. In recent years, WBRT has been used less frequently due to technological advances and to avoid cognitive decline after treatment.

Some reports have explored the prognostic factors of BM, identifying a low Karnofsky Performance Status,^10^, ^11^ a high number of BMs,^10^ extracranial metastasis,^10^ high‐performance status,^10^ not receiving chemotherapy,^1^, ^2^, ^12^ non‐WBRT,^2^ non‐SRS,^2^ non‐metastasectomy,^1^, ^2^, ^3^, ^12^ old age,^8^ hemorrhaging tumor,^1^ short duration from the initial diagnosis to BM,^7^ high histological grade,^2^, ^10^ and non‐alveolar soft part sarcoma (ASPS)^3^, ^5^, ^8^ as poor prognostic factors. However, owing to the rareness of tumor and severe poor prognosis, limited clinical data and oncological outcomes regarding BM of sarcoma are available. Over the past few decades, improvements in imaging findings, such as computed tomography (CT) and magnetic resonance imaging (MRI), have improved the accuracy of diagnosis of BM. The improvement of the overall survival in patients with sarcoma due to recent advances including multi‐disciplinary treatment will necessarily increase the incidence of BM.^7^, ^17^ Therefore, the accumulation of experience concerning the diagnosis and treatment of BM of sarcoma is desirable.

With regard to classical systemic therapies, the ability to penetrate the brain–blood barrier is reportedly critical for treating BM. The landscape of treatment of BM from other cancers has evolved in recent years. Among developed modalities, tyrosine kinase inhibitors, such as sunitinib, sorafenib, and pazopanib, have drastically changed the strategy of BM treatment for various histological subtypes of malignant tumors. In addition, Takahashi et al. showed that eribulin was able to penetrate brain tumors.^18^ Among these drugs, pazopanib and eribulin have already been approved for the treatment of advanced soft tissue sarcoma.^19^ However, in recent years, SRS came to be widely used to treat BM instead of WBRT. In addition, it is important to analyze clinical outcomes based on the latest WHO classification and latest staging system, as the classification of bone and soft tissue sarcoma has changed with the development of molecular analysis approaches in recent years.

In the present study, we retrospectively reviewed sarcoma patients with BM diagnosed and treated in a large cohort of patients with sarcoma at our facility database. We report the detailed clinical characteristics and clinical presentation of BM from sarcomas based on the latest histopathological classification.

## 2 MATERIALS AND METHODS

### 2.1 Patients and approaches

After obtaining approval from the institutional review board (ethical review number. 2021‐346), The study was conducted in accordance with the Declaration of Helsinki. Informed consent was obtained from all participants included in this study.

In all cases, the definitive diagnosis of primary sarcoma was confirmed by a pathological examination. Thirty‐two patients diagnosed with BM who were retrieved via medical records between 2006 and 2021 among 3133 bone and soft tissue sarcoma patients were included in our study. Cases with cranial metastases and primary brain sarcoma were excluded. Clinicopathological characteristics, including the sex, age at the diagnosis of BM, histological subtype, location of tumor, stage (The American Joint Committee on Cancer [AJCC], 8th),^20^ treatment (before and after BM), existence of extracranial metastases (lung and/or bone, other vital organs), clinical symptoms concerning BM, duration until the diagnosis of primary sarcoma and BM, quantity of metastases (solitary or multiple), and oncological outcome, were retrospectively collected.

### 2.2 Statistical analyses

The BM‐free survival (BMFS) was defined as the interval from the date of the diagnosis of primary sarcoma to the date of BM, and the post‐BM survival (PBMS) was determined from the date of BM to the date of tumor death or last follow‐up. The duration between initial metastasis and BM was defined from the date of extracranial metastasis to the date of BM. The mean duration was defined as the cut‐off point for the duration between initial metastasis and BM. Survival curves were calculated using the Kaplan–Meier curve for each factor. Differences in survival were assessed by the log‐rank test, and statistical significance was defined as *p* < 0.05.

Data analyses were performed using the JMP statistical software package (version 14.0.0; SAS Institute Inc.).

## 3 RESULTS

### 3.1 Clinicopathological results

**FIGURE 1:**
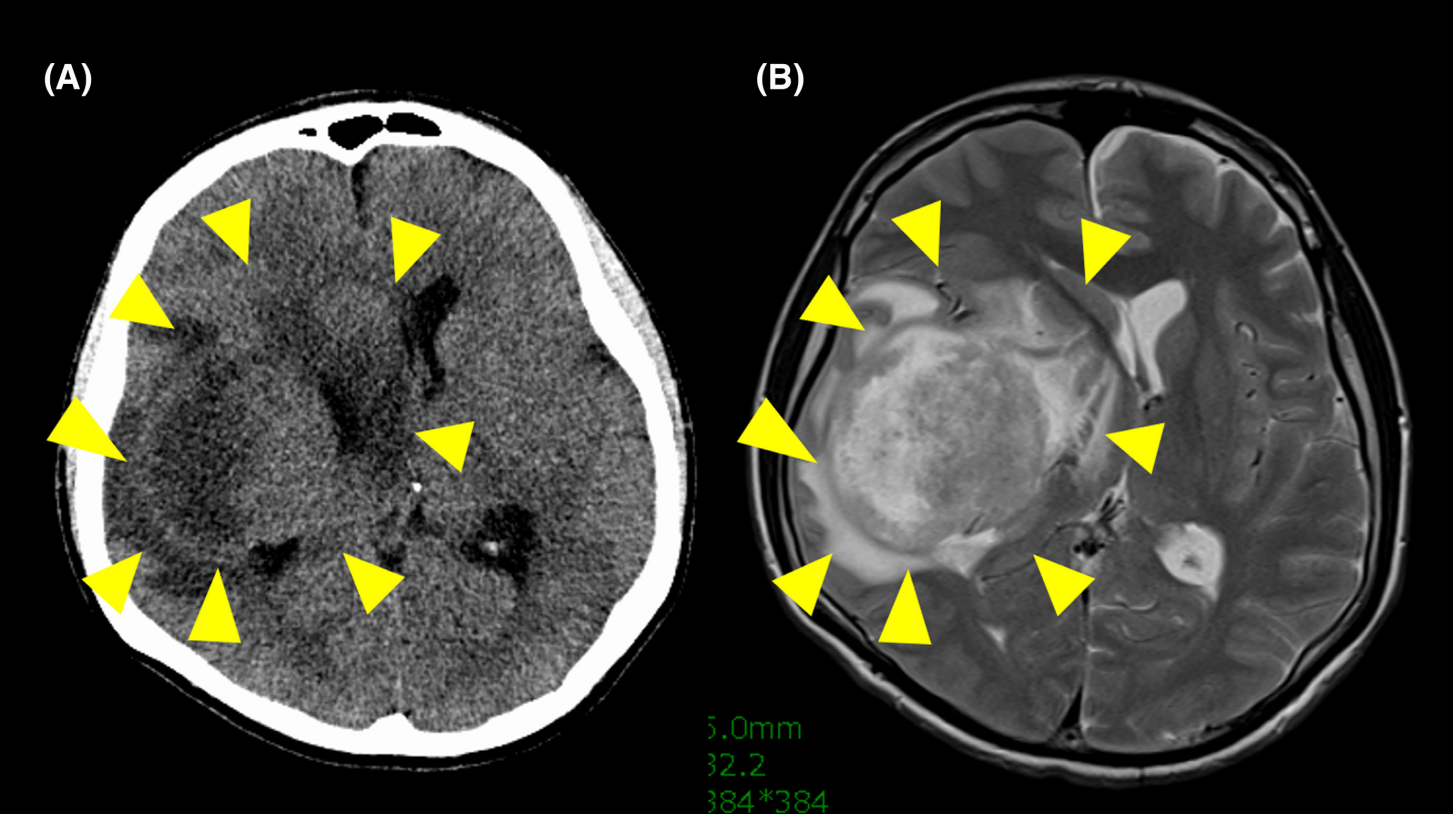
Representative brain computed tomography (A) and T2‐weighted magnetic resonance imaging (B) of brain metastasis of sarcoma (malignant peripheral nerve sheath tumor). A brain tumor in the right cerebral hemisphere (arrow) and a midline shift can be seen.

The clinicopathological characters of the subjects are shown in Table 1. The representative radiological findings are shown in Figure 1. The age at the diagnosis of BM ranged from 10 to 66 (median: 39) years old. There were 17 males and 15 females among the patients with BM. Focused on primary sarcoma, tumors arose in the bone (*n* = 7) and soft tissue (*n* = 25). The histological subtypes of primary tumors are also shown in Table 1. ASPS (*n* = 8), undifferentiated pleomorphic sarcoma (UPS, *n* = 8; two bone and six soft tissue), malignant peripheral nerve sheath tumor (*n* = 4) and Ewing sarcoma (*n* = 3, all cases from soft tissue origin) were common histological subtypes.

**TABLE 1:** Clinicopathological characteristics of BM of sarcoma.

No.	Histology	Age (years)	Sex	Before BM							After BM					
				Primary site	AJCC stage**	Lung meta	Bone meta	Other meta	Treatment	BMFS (months)	Symptom	Number of BM	Treatment	Regimen	PBMS (months)	Outcome
1	AS	63	M	Lung	‐	Present	Absent	Absent	N/A	0	N/A	N/A	Chemotherapy		0.5	DOD
2	AS	21	M	Retroperitoneum	‐	Absent	Absent	Absent	Chemotherapy	N/A	Headache, nausea, visual disturbance	Solitary	Surgery + chemotherapy	Topotecan+CPA	2	DOD
3	ASPS	38	F	Shoulder	IV	Present	Absent	Absent	Surgery	19	Limb paralysis	Multiple	SRS		19	AWD
4	ASPS	20	M	Buttock	IV	Present	Absent	Absent	Chemotherapy	20	Headache	Solitary	SRS		20	DOD
5	ASPS	33	M	Abdominal wall	IIIA	Present	Present	Present	Surgery	5	N/A	Multiple	SRS		5	AWD
6	ASPS	46	M	Shoulder	IV	Present	Present	Absent	Surgery + chemotherapy	28	N/A	N/A	SRS		28	DOD
7	ASPS	40	M	Buttock	IV	Present	Absent	Present	Surgery	5	Hoarseness	Solitary	Chemotherapy	ADR + DTIC	5	DOD
8	ASPS	21	F	Thigh	IIB	Present	Absent	Absent	Surgery + chemotherapy	2	None	Multiple	Surgery + chemotherapy	Sunitinib	2	DOD
9	ASPS	31	F	Thigh	IIIB	Absent	Absent	Absent	Surgery	N/A	Double vision	N/A	Surgery + chemotherapy + SRS	PAZ	91	AWD
10	DDLS	43	M	Thigh	IIIB	Present	Absent	Present	Surgery + chemotherapy	1	Hyperalgesia, dysgeusia	Solitary	3D‐CRT		1	DOD
11	Epithelioid sarcoma	54	F	Hand	II	Present	Present	Present	Surgery + chemotherapy	3	None	Solitary	Surgery + chemotherapy + WBRT	ADR	3	DOD
12	Ewing	25	M	Pelvic cavity	IV	Present	Absent	Present	Surgery + chemotherapy	0	Limb paralysis	Solitary	None		0.5	DOD
13	Ewing	36	M	Mediastinum	IV	Present	Present	Present	Chemotherapy	5	Headache	Solitary	Surgery + chemotherapy + WBRT	VCR + ADR + CPA/IFO + VP‐16	5	DOD
14	Ewing	25	F	Nasal cavity	IIA	Absent	Absent	Absent	Surgery + chemotherapy	N/A	Fascial pain	Solitary	Surgery + chemotherapy	N/A	36	DOD
15	High‐grade sarcoma of bone	31	F	Tibia	IV	Present	Present	Absent	Chemotherapy	3	Limb paralysis	Multiple	Chemotherapy + WBRT	ADR + CDDP	3	DOD
16	MPNST	41	F	Retroperitoneum	IV	Present	Absent	Absent	Surgery	2	Limb paralysis	Solitary	None		2	AWD
17	MPNST	52	M	Thigh	IV	Present	Absent	Absent	Surgery + chemotherapy	0	Disturbance of consciousness	Multiple	None		0.5	DOD
18	MPNST	40	F	Popliteal fossa	IIIA	Present	Present	Present	Surgery + chemotherapy	22	None	Solitary	Chemotherapy	ADR + IFO, IFO + VP16	22	DOD
19	MPNST	33	M	Inguinal lesion	IIIA	Present	Present	Present	Surgery + chemotherapy	3	Headache	Solitary	Surgery + chemotherapy +WBRT	GEM+DOC	3	DOD
20	OS	10	F	Femur	IIB	Present	Absent	Absent	Surgery + chemotherapy	14	Headache, visual disturbance	Multiple	Chemotherapy	TMZ + VP‐16	14	DOD
21	OS	30	F	Humerus	IIB	Present	Absent	Present	Surgery + chemotherapy	0	N/A	Multiple	WBRT		0.5	DOD
22	RMS	38	M	Lung	IV	Present	Present	Absent	Chemotherapy	1	Headache	Solitary	WBRT		1	DOD
23	RMS	41	F	Buttock	IIIB	Present	Present	Present	Chemotherapy	1	Headache, numbness	Multiple	Surgery + chemotherapy + WBRT	IFO	1	DOD
24	UPS	66	M	Back	IV	Present	Present	Present	Surgery + chemotherapy	1	Limb paralysis	Multiple	WBRT		1	DOD
25	UPS	45	F	Buttock	IIIA	Present	Present	Present	Surgery + chemotherapy	4	Visual disturbance	Multiple	Surgery + chemotherapy + WBRT	IFO + VP16	4	AWD
26	UPS	64	F	Femur	IIB	Present	Absent	Present	Surgery + chemotherapy	5	Headache, nausea	Solitary	SRS		5	DOD
27	UPS	38	M	Humerus	IIB	Present	Absent	Present	Surgery + chemotherapy	9	Limb paralysis	Solitary	Surgery +3D‐CRT		9	DOD
28	UPS	48	M	Neck	IV	Present	Absent	Absent	Chemotherapy	10	Headache	Multiple	SRS		10	DOD
29	UPS	57	F	Knee	IIIA	Present	Absent	Present	Surgery + chemotherapy	20	Limb paralysis	Multiple	Surgery + chemotherapy +3D‐CRT	ERB	7	DOD
30	UPS	65	M	Chest wall	II	Present	Absent	Absent	Surgery + chemotherapy	13	Headache	Multiple	Surgery + WBRT		4	AWD
31	ASPS	36	M	Back	IV	Present	Present	Present	N/A	N/A	Headache, verb disturbance, convulsion	Multiple	N/A		N/A	N/A
32	UPS	57	F	Thigh	IV	Absent	Absent	Present	N/A	N/A	Headache, nausea	Multiple	N/A		N/A	N/A

Abbreviations: 3D‐CRT, three‐dimensional conformal radiation therapy; ADR, adriamycin; AS, angiosarcoma; ASPS, alveolar soft part sarcoma; AWD, alive with disease; BM, brain metastasis; BMFS, brain metastasis‐free survival; CDDP, cisplatin; CPA, cyclophosphamide; DDLS, dedifferentiated liposarcoma; DOC, docetaxel; DOD, died of disease; DTIC, dacarbazine; ERB, eribulin; F, female; GEM, gemcitabine; IFO, ifosfamide; M, male; meta, metastasis; MPNST, malignant peripheral nerve sheath tumor; N/A, not available; O, osteosarcoma; PAZ, pazopanib; PBMS, post‐brain metastasis survival; RMS, rhabdomyosarcoma; SRS, stereo radiosurgery; TMZ, temozolomide; UPS, undifferentiated pleomorphic sarcoma; VCR, vincristine; VP‐16, etoposide; WBRT, whole‐brain radiotherapy.

* Patients of No. 31 and 32 were not followed up continuously.

** AJCC: The American Joint Committee on Cancer, 8th.

Clinical outcome data were available for 30 patients, with follow‐up durations ranging from 0.5 to 321 (median: 27) months after the diagnosis of the primary tumor. Twenty‐four patients died from disease, and six patients were alive at the end of the follow‐up. Twenty‐two patients underwent surgery, and 25 were treated with chemotherapy before BM. Twenty‐nine patients had BM preceded by extracranial metastasis, including lung metastasis (*n* = 27), bone metastasis (*n* = 12), and other distant metastasis (*n* = 17). The mean duration between initial metastasis and BM was 43 (range: 0–316) months.

### 3.2 Clinical characteristics of BM

**FIGURE 2:**
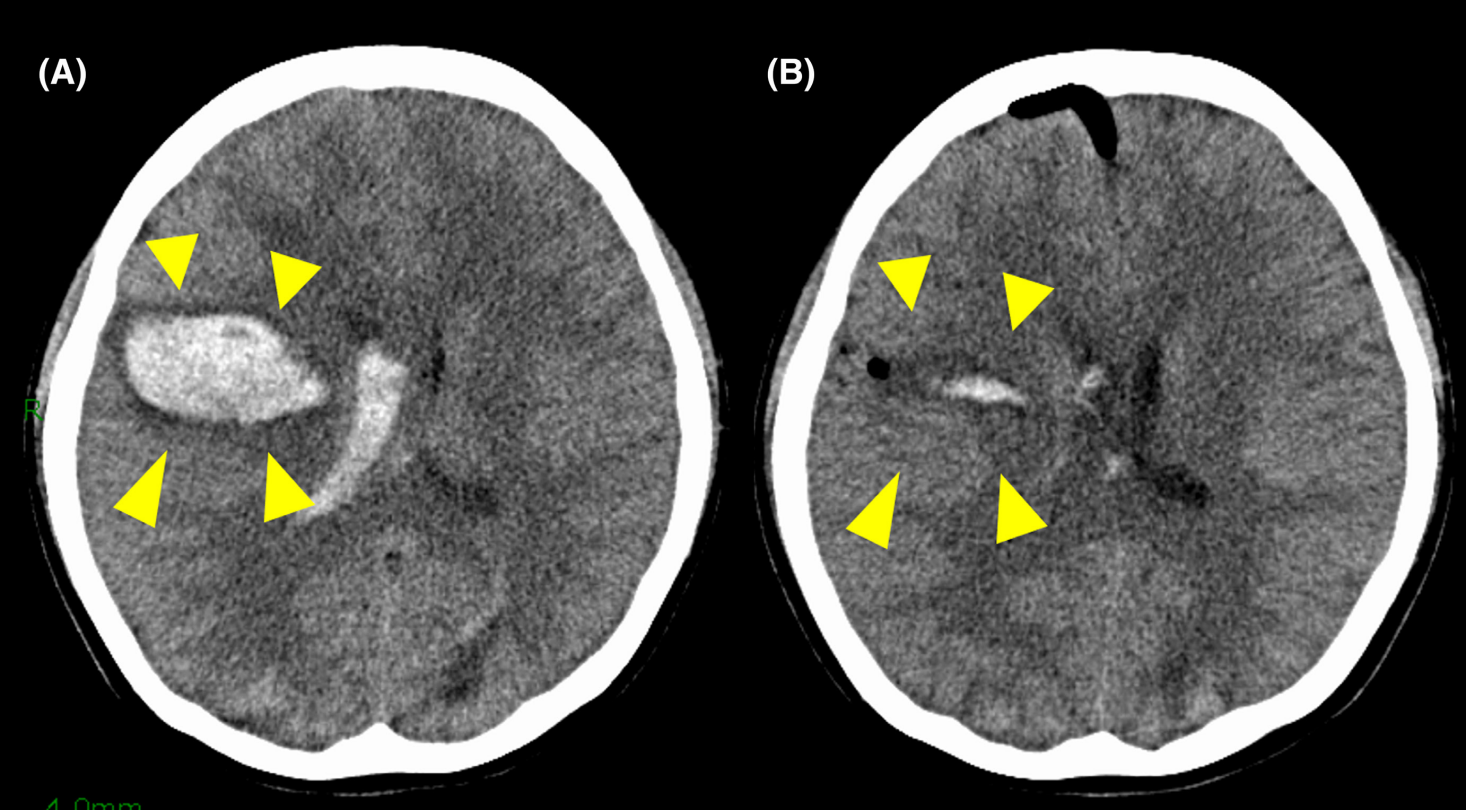
A patient who developed cerebral hemorrhaging after the administration of sunitinib for brain metastasis of alveolar soft part sarcoma. Preoperative non‐enhanced computed tomography (CT) showed cranial hemorrhaging in the right hemisphere (arrow) with a mild midline shift (A). Postoperative CT performed 5 days after craniotomy revealed mild residual bleeding (arrow) (B). The histological examination of the removed hematoma showed the presence of a tumor (data not shown).

The clinical characteristics of BM and treatment for BM are summarized in Table 1. The common symptoms regarding BM were as follows: headache (*n* = 11), limb paralysis (*n* = 7), visual disturbance (*n* = 3), and nausea (*n* = 3). Fifteen patients had multiple BMs, and 14 had solitary BM; the number of BMs could not be determined in 3 cases. After the diagnosis of BM, 27 patients received treatment in some form, including chemotherapy in 15 patients, surgery in 12, and radiotherapy in 20 (WBRT [*n* = 9], stereotactic radiosurgery [SRS; *n* = 7], three‐dimensional conformal radiation therapy [*n* = 4]). Eight patients received both surgery and radiotherapy (WBRT [*n* = 6], SRS [*n* = 1], 3D‐CDT [*n* = 1]). Seven of eight patients also underwent chemotherapy. Of note, among patients treated with chemotherapy, two with metastatic ASPS were treated with sunitinib, a multi‐targeted tyrosine kinase inhibitor. After the administration of sunitinib, both patients suffered cerebral hemorrhaging. One patient (Figure 2) underwent craniotomy, and the other patient received the best supportive care.

### 3.3 Oncological outcomes in patients with BM

**FIGURE 3:**
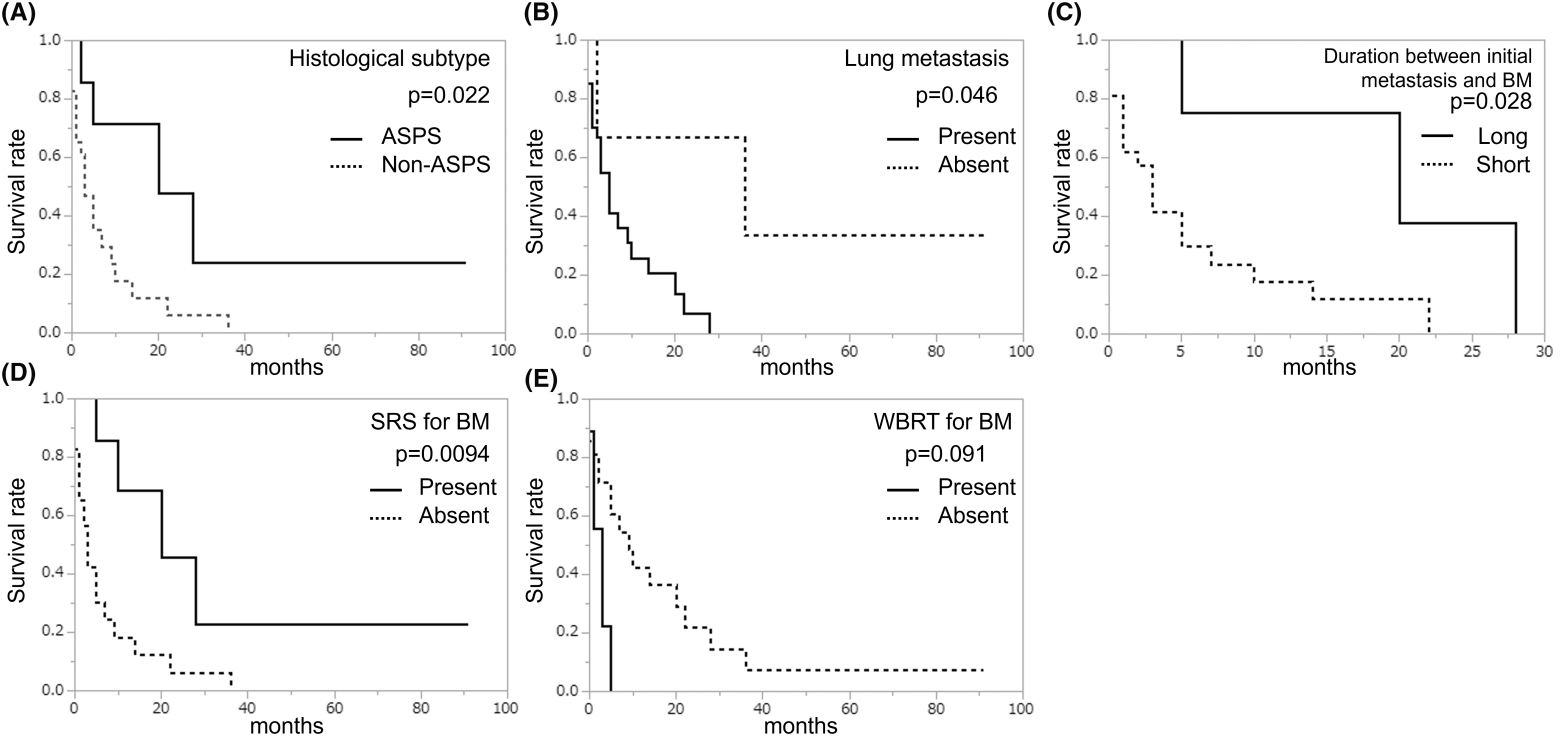
Kaplan–Meier survival curves of histology (A), lung metastasis (B), duration between initial metastasis and BM (C), treatment for BM with stereotactic surgery (D), treatment for BM with whole‐brain radiotherapy (E) and the post‐BM survival (Log‐rank test). ASPS, alveolar soft part sarcoma; BM, brain metastasis; SRS, stereotactic surgery; WBRT, whole‐brain radiotherapy.

We assessed the prognostic significance concerning the PBMS for clinicopathological characteristics and treatment after BM using a Kaplan–Meier survival analysis (Table 2). The median PBMS was 4 (range: 0.5–91) months. ASPS (*p* = 0.022) (Figure 3A), no lung metastasis (*p* = 0.046) (Figure 3B), a long duration between initial metastasis, and the diagnosis of BM (*p* = 0.028) (Figure 3C) and SRS for BM (*p* = 0.0094) (Figure 3D) were significantly associated with a favorable prognosis. In contrast, the cases that received only WBRT had a significantly worse prognosis than those that received other treatments instead of WBRT (*p* = 0.0091) (Figure 3E). Due to the limited case numbers, a multivariate analysis was not performed.

**TABLE 2:** Results of univariate analysis for post‐brain metastasis survival associated with clinicopathological characteristics.

Parameter	*N*	Median (mos)	95% CI	1‐year PBMS (%)	*p* Value^a^
Age (years)					
<50	23	9	2–20	39	0.068
≥50	7	3	0–7	0	
Sex					
Male	16	4	1–10	17.8	0.12
Female	14	7	2–36	44	
Site					
Soft tissue	24	5	2–20	35	0.21
Bone	6	4	0–14	16	
Histological variant					
Non‐ASPS	23	3	1–7	17	0.022*
ASPS	7	20	2‐NA	71	
ECM					
Lung					
Absent	3	36	2‐NA	66	0.046*
Present	27	5	1‐10	26	
Bone					
Absent	19	7	1–14	31	0.71
Present	11	3	1–22	34	
Other					
Absent	15	14	1–28	50	0.076
Present	15	5	1–7	10	
Number					
Solitary	14	5	1–9	23	0.87
Multiple	13	7	1–14	26	
BM treatment					
Any					
Absent	3	0	0	0	0.064
Present	27	5	2–14	32	
Chemotherapy					
Absent	15	9	0–20	30	0.79
Present	15	5	2–14	30	
SRS					
Absent	23	3	1–7	18	0.0094*
Present	7	20	5‐NA	68	
WBRT					
Absent	21	9	2–22	42	0.0091*
Present	9	3	0‐5	0	
Duration between initial metastasis and BM					
Short	24	3	1–7	20	0.020*
Long	6	28	5‐NA	80	

Abbreviations: ASPS, alveolar soft part sarcoma; BM, brain metastasis; CI, confidence interval; ECM, extracranial metastasis; NA, not available; PBMS, post‐brain metastasis survival; SRS, stereotactic radiosurgery; WBRT, whole‐brain radiotherapy.

^a^ Log‐rank test.

* Siginificant difference.

### 3.4 Prognostic factors for the BMFS

**FIGURE 4:**
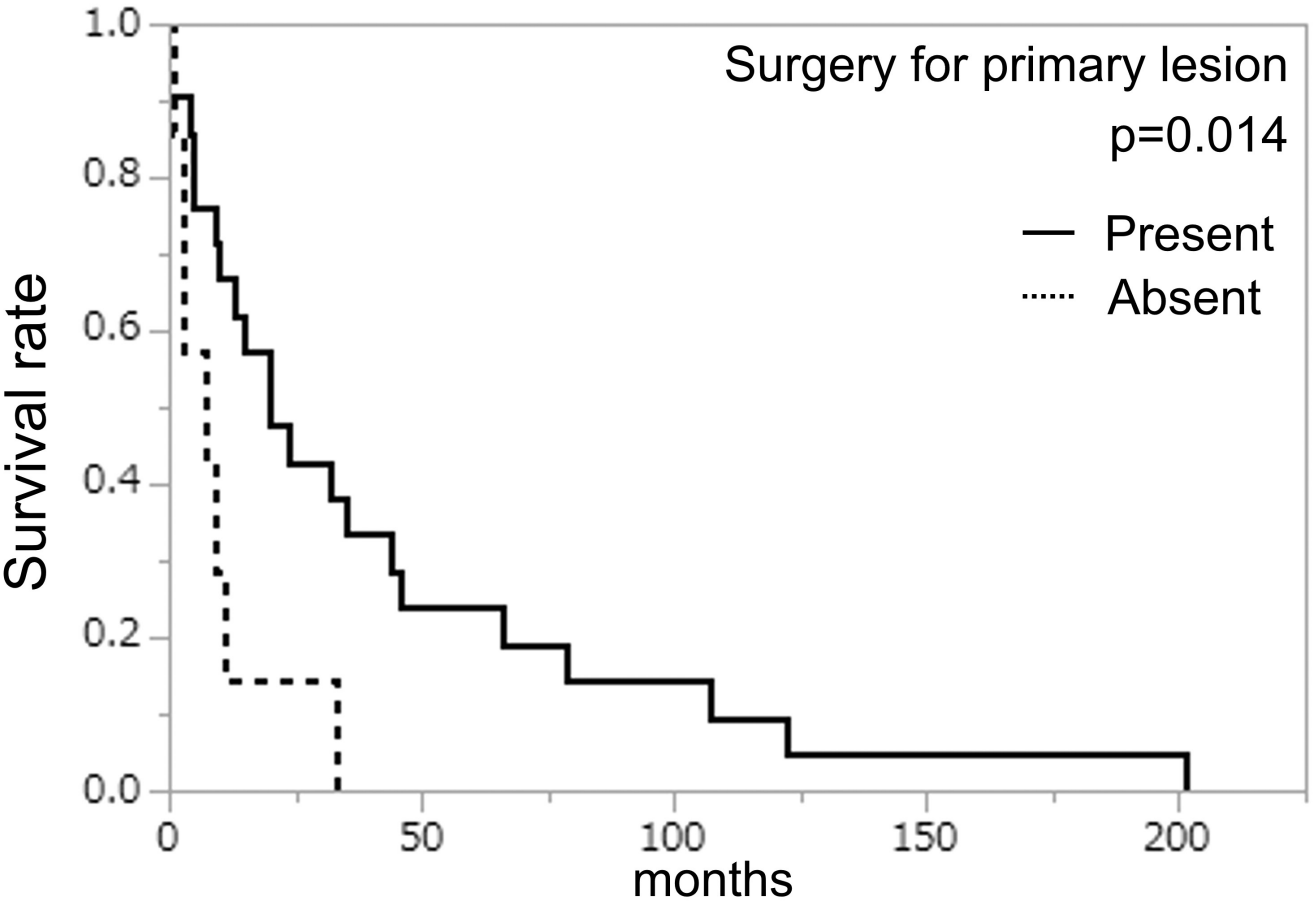
Kaplan–Meier survival curves of surgery before brain metastasis illustrate the brain metastasis‐free survival (Log‐rank test).

The BMFS ranged from 0 to 316 (median: 11) months. We assessed the prognostic significance concerning the BMFS for clinicopathological characteristics and treatment before BM using a Kaplan–Meier survival analysis (Table 3) The cases that received surgery for the primary tumor had a significantly longer BMFS than those that did not undergo surgery (*p* = 0.014) (Figure 4).

**TABLE 3:** Results of univariate analysis for the BMFS associated with clinicopathological characteristics.

Parameter	N	Median (mos)	95% CI	1‐year BMFS (%)	*p* Value^a^
Age (years)					
<50	23	13	5–35	50	0.89
≥50	7	13	0–20	57	
Sex					
Male	16	10	4–24	40	0.73
Female	14	20	1–44	64	
Site					
Soft tissue	24	11	5–33	47	0.57
Bone	6	22	1–44	66	
Histological variant					
Non‐ASPS	23	10	4–20	43	0.059
ASPS	7	39.5	9–201	85	
Treatment before BM					
Surgery					
Absent	7	7	1–11	14	0.014*
Present	22	20	9‐44	66	
Chemotherapy					
Absent	5	50	0–201	60	0.15
Present	24	54	5–32	52	

Abbreviations: ASPS, alveolar soft part sarcoma; BM, brain metastasis; BMFS, brain metastasis‐free survival; CI, confidence interval.

^a^ Log‐rank test.

* Significant difference.

## 4 DISCUSSION

In the general clinical course of sarcoma, BM is a relatively exceptional event. In previous studies, the incidence of BM was reported to be 0.6%–3.2% among patients with sarcoma.^3^, ^4^, ^6^, ^12^, ^15^ In our study, the incidence of BM was 1.2%, which was consistent with previous studies. Chief complaints related to BM have also been discussed in some papers.^1^, ^2^, ^3^, ^11^, ^12^ The most common symptom related to BM was a headache according to the literature, with this symptom seen in 11%–48% of patients.^1^, ^2^, ^3^, ^11^ In our study as well, the most common symptom of BM was a headache, which is consistent with previous studies. In our cohort, 11.5% of patients had been incidentally detected to have BM. Previous studies showed that detecting BM was chance findings in 0%–12% of sarcoma patients.^1^, ^2^, ^3^, ^11^ It has been reported that BM of sarcoma was preceded by extracranial metastasis, with the most common site of metastasis being the lung (incidence rate: 51%–100%).^2^, ^6^, ^9^, ^11^, ^12^ Ultimately, 84% of our patients also developed lung metastasis.

In the previous literature, the most common histological subtype of primary sarcoma in patients with BM showed wide variation, including leiomyosarcoma,^2^, ^7^ osteosarcoma,^3^, ^11^ UPS^1^ and ASPS.^15^ The most common histological subtype in our study was ASPS. Although ASPS frequently metastasizes to distant organs,^21^, ^22^ the metastatic lesion is usually indolent.^23^, ^24^ Evidence concerning the effectiveness of periodic imaging examinations to detect BM among patients with sarcoma has not been established.^3^, ^23^ However, for other malignancies, such as breast cancer^25^ and malignant melanoma,^26^ six‐monthly periodic imaging analyses for screening can aid in the early detection of BM. We, therefore, recommend periodic head CT or MRI in addition to routine examinations in ASPS patients with other distant metastases.

BM from sarcoma is preceded by metastases to extracranial sites and is considered a late stage of the disease. In the present study, the median BMFS was 14 months. In previous studies, the median BMFS ranged from 16 to 37 months.^1^, ^2^, ^3^, ^7^, ^9^, ^11^, ^12^ Chou et al. investigated the timing of the development of BM among patients with osteosarcoma and soft tissue tumors and found a discrepancy in the timing of BM between these two tumor types.^3^ They showed that the incidence of BM plateaued 3 years after the diagnosis of osteosarcoma, whereas it continued to increase over time for soft tissue tumors.^3^ In our study, the longest BMFS was 316 months, and the patients with ASPS tended to have a longer BMFS than those with other tumors. These results suggest that patients with ASPS may develop BM long after the diagnosis of the primary tumor. Therefore, ASPS patients should receive careful long‐term follow‐up with periodic radiological examinations.

The prognosis for BM is abysmal.^1^, ^2^, ^3^, ^4^, ^7^, ^9^, ^10^, ^11^ The median PBMS in our study was 4 months, which is consistent with previous studies (range: 1.6–7.5 months).^1^, ^2^, ^3^, ^5^, ^7^, ^12^ We detected that ASPS, no lung metastasis, a long duration between initial metastasis, and the diagnosis of BM and SRS for BM were significantly associated with a favorable prognosis. Previous studies^3^, ^5^, ^8^ reported that ASPS significantly had a better prognosis than non‐ASPS, which was consistent with our present findings. Furthermore, as in previous studies,^7^, ^9^ a shorter BMFS was significantly associated with a poor prognosis, indicating the rapid progression of the disease.

Although the prognosis for patients with BM from sarcoma is poor, several treatment options have been considered. Malouf et al. performed the largest retrospective analysis of ASPS patients with BM to date and explored the potential efficacy of angiogenesis therapy.^9^ Classically, various clinical trials against advanced ASPS have been performed,^27^, ^28^ describing the response to sunitinib, a tyrosine kinase inhibitor in advanced ASPS.^9^, ^29^, ^30^ Sunitinib can likely penetrate the blood–brain barrier and is expected to exert a potential anti‐tumor effect on BM,^31^ whereas classical chemotherapy, such as doxorubicin, which is a key drug for the treatment of sarcoma, has shown poor penetration of the blood–brain barrier.^32^ We treated two patients with BM from ASPS with sunitinib, with both responding well but suffering cerebral hemorrhaging. Peritumoral brain hemorrhaging was reported to be correlated with an extremely poor prognosis.^1^ Another study evaluated the efficacy and safety of sunitinib among patients with unresectable or metastatic ASPS. Hemorrhaging was found in 35% of those patients as an adverse event.^30^ Furthermore, in renal carcinoma, a high incidence of cerebral hemorrhaging in BM patients treated with sunitinib was reported.^33^ Since ASPS is a richly vascularized tumor, the risk of cerebral hemorrhaging due to treatment effects should be noted when we administer sunitinib to patients with BM. Radiation therapy may be considered for the prevention of intracranial hemorrhaging before the administration of sunitinib. Of note, there was a case report of a patient with BM from renal cell carcinoma who had a prolonged survival following treatment with pazopanib^34^, which is a multi‐targeted tyrosine kinase inhibitor like sunitinib. However, another report showed a reduced effectiveness of pazopanib for BM from ASPS.^35^ Furthermore, according to animal experiments, the drug delivery of pazopanib was hindered by the blood–brain barrier.^36^ Therefore, whether or not pazopanib is similarly effective in patients with BM remains controversial. Pazopanib has been approved in Japan as a second‐line treatment for patients with advanced soft tissue sarcoma.^19^ It is necessary to investigate the effectiveness of pazopanib against BM of sarcoma. Eribulin, which is also used to treat advanced cases of soft tissue sarcoma, can penetrate brain tumors,^18^ suggesting it as a promising treatment option for patients with BM of sarcoma.

Although our study failed to demonstrate a significant effect of surgery for BM on the PBMS, some studies have mentioned that surgery for BM improved the PBMS.^1^, ^2^, ^4^, ^7^, ^12^ In contrast, SRS, such as gamma‐knife, is considered a local treatment option for BM. In other malignancies such as lung cancer, breast cancer, melanoma, colorectal cancer, and renal cell cancer, a drastic change in the initial treatment of BM has been observed over time.^37^ The number of SRS procedures increased over that of surgery for the treatment of BM in sarcomas.^37^ In addition, the median PBMS has significantly improved over time.^37^ The generalization of SRS may have improved the median OS. The effectiveness of SRS has also been reported in other malignancies, such as renal cell carcinoma^38^ and malignant melanoma.^39^ Regarding sarcomas, SRS was performed in 5.9%–15% of patients with BM.^2^, ^3^, ^7^, ^10^ In our study, SRS was performed in 25% of patients, showing a higher frequency than in previous reports, and was significantly correlated with a better prognosis. However, Liu et al. pointed out the possibility of recurrence after SRS treatment and emphasized the importance of follow‐up with imaging examinations.^40^ WBRT is a treatment option for BM and is reported to improve the prognosis of patients with BM.^2^ In contrast to that study, the patients in our study treated with WBRT for BM had a worse prognosis than those who did not receive WBRT, possibly because patients who received WBRT had more severe disease progression with no other treatment options.

Several limitations associated with the present study warrant mention. First, the sample sizes were relatively small due to the rarity of sarcoma and the incidence of BM. However, we retrospectively reviewed sarcoma patients with BM diagnosed and treated in a very large cohort of patients with sarcoma at our facility database. Furthermore, all histological diagnoses were based on the newest WHO classification. Second, there has been no imaging evaluation of the cranial area for patients with asymptomatic BM. As a result, the number of BM may be underestimated.

In conclusion, we retrospectively investigated the clinicopathological characteristics and prognosis of bone and soft tissue sarcoma patients with BM. In the current study, the most common symptom was a headache. Favorable prognostic factors related to BM were ASPS sarcoma, absence of lung metastasis, a longer duration between initial metastasis, and the diagnosis of BM and SRS for BM. Although the prognosis after BM remains poor in patients with sarcoma, the further accumulation of experience concerning newly developed treatment modalities, such as tyrosine kinase inhibitors and SRS, for BM of sarcoma is desirable.

## AUTHOR CONTRIBUTIONS

**Yu Toda:** Conceptualization (equal); data curation (lead); investigation (lead); writing – original draft (lead). **Eisuke Kobayashi:** Conceptualization (lead); project administration (equal); writing – review and editing (lead). **Daisuke Kubota:** Conceptualization (equal); data curation (lead); investigation (supporting). **Yasuji Miyakita:** Data curation (supporting); investigation (lead). **Yoshitaka Narita:** Conceptualization (equal); project administration (supporting); writing – review and editing (supporting). **Akira Kawai:** Conceptualization (equal); project administration (lead); writing – review and editing (equal).

## CONFLICT OF INTEREST STATEMENT

None.

## Data Availability

Data sharing is not applicable to this article as no new data were created or analyzed in this study.
